# Fire Ant Alate Wing Motion Data and Numerical Reconstruction

**DOI:** 10.1673/031.010.1901

**Published:** 2010-03-15

**Authors:** L. Gui, T. Fink, Z. Cao, D. Sun, J.M. Seiner, D.A. Streett

**Affiliations:** ^1^ NCPA, University of Mississippi, University, MS 38677; ^2^ USDA-ARS, Biological Control of Pests Research Unit, Stoneville, MS 38776

**Keywords:** insect flight, stereo imaging, high-speed imaging, computational fluid dynamics, *Solenopsis richteri*, *Solenopsis invicta*

## Abstract

The wing motions of a male and a female fire ant alate, which beat their wings at 108 and 96 Hz, respectively, were captured with a stereo imaging system at a high frame rate of 8,000 frames per second. By processing the high-speed image frames, the three-dimensional wingtip positions and the wing surface orientation angles were determined with a high phase resolution, i.e. 74 and 83 phases per period for the male and the female, respectively. A numerical reconstruction of the stereo wingbeat images demonstrated that the data collected described almost all the details of the wing surface motion, so that further computational fluid dynamic simulations are possible for fire ant alate flight.

## Introduction

Imported fire ants are major pests of urban, agricultural and wildlife areas (Adams 1986; Allen et al. 1995; Boman et al. 1995; Taber 2000; Wojcik et al. 2001). The black imported fire ant, *Solenopsis richteri* Forel (Hymenoptera: Formicidae), was introduced first in Mobile, Alabama about 1918 and the red imported fire ant, *Solenopsis invicta* Buren, was introduced between 1933–1945 in the same port. Imported fire ants can now be found throughout the Southeast and in scattered localities in the extreme Southwest United States (Callcott and Collins 1996; Taber 2000; Tschinkel 2006). An important way for imported fire ants to disperse is the mating flight (Markin et al. 1971; Morrill 1974). The most comprehensive studies of fire ant mating flights were by Markin et al. (1971). They found an average yearly expansion of the *S. richteri* range of ∼3–5 miles. Using airplanes with retractable nets and passive black light traps at different heights on a TV transmission tower, they found females up to 800 feet high and males to 1000 feet. They believed males did not swarm over a swarm marker like most hymenoptera, but rather flew in a layer 200– 500 feet high. Males flew from late morning or early afternoon to near dusk, while females remained aloft 30 minutes or less, and 95% returned inseminated. No direct observations of swarming or mating were made and apparently none have been made by others. Vogt et al. (2000) investigated flight energetics and dispersal capability of *S. invicta* alates by recording flight distance and speed in a flight mill and recorded respiration in closed-system and flow-through flight respirometers. In the absence of wind, *S. invicta* female alates flew less than 5 km. They also recorded wingbeat frequencies but did not study the detailed motion of the wings. In mosquitoes and Chironomidae it is known that frequencies/tones generated by wingbeat are important for attraction between males and females in swarms (Roth 1948; Wishart and Riordan 1959; Belton and Costello 1979; Ogawa and Sato 1993; Fyodorova and Azovsky 2003; Gibson and Russell 2006). The present study using high-speed video is the first of a detailed investigation of fire ant flight that attempts to understand wingbeat induced sound waves and associated air flows that may be important in communication and mate localization.

High-speed video techniques have been widely used to capture wing motion of bird and insect flights (Norris 2001; Sudo et al. 2001, 2005; Dalton 2002; Gorman 2003). Recent advances in high-speed imaging technique make it possible to capture insect wing motion not only at high phase resolution but also at high digital resolution, e.g. the Photron Ultima APX high-speed camera used in our lab is capable of capturing partial frames of 1024×256 pixels at 8,000 frames per second (fps). High digital resolution and high phase resolution together enable a precise analysis of the wing motion within a wingbeat period. However, at least two images from different view angles, e.g. provided by a stereo imaging system, are necessary to determine the three dimensional shape and orientation of the wing surface. A stereo imaging system requires at least two cameras that view an object from two different directions, and in an ideal situation, these two view directions should be perpendicular to each other, (Sudo et al. 2001, 2005). Since one advanced high-speed camera is already very expensive for most research laboratories, building a stereo imaging system with two high-speed cameras is usually not possible. Therefore, a stereo high-speed imaging system was constructed using a single high-speed camera for the fire ant alate tests. One important reason for conducting the stereo high-speed imaging tests is to collect the necessary data for computational fluid dynamics (CFD) simulations of the fire ant alate flight. CFD has been widely used to solve fluid flow problems and the accompanied acoustic problems in engineering areas since the invention of computers, and it was recently used to solve problems of insect flight (Kazuhiro 2000; Sane 2003; Mao and Gang 2003; Cheng et al. 2006)

In the present work, stereo high-speed image frames were acquired for wing motions of many black and red imported hybrid fire ant males and females that were tethered with fine metal wires and stimulated to fly with a mini blower in the lab. Two candidates from the males and females, respectively, were selected for further detailed analyses to determine the three-dimensional wingtip positions and the wing surface orientation angles. The resulting fire ant wing motion data were verified with a numerical reconstruction of the stereo image frames. This allowed the development of a numerical model for CFD simulation. Since some of the near field sound waves were simultaneously measured with the wingbeat motion test, the wingbeat motion data was be used to explain some features of the sound waveforms.

**Figure 1.**
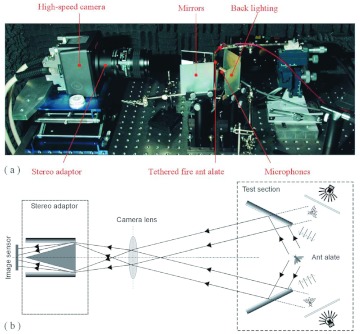
Experimental setup: (a) photo of the stereo high-speed imaging system; (b) schematic illustration of the stereo high-speed imaging system. High quality figures are available online.

## Materials and Methods

### Specimen collection and rearing in the laboratory

Colonies of *S. richteri* were sampled for alates in Lafayette County, Mississippi including the University of Mississippi Oxford campus, Holly Springs National Forest. University of Mississippi field station and other localities. A limited number of ants were dug up if alates were present, placed in trays with sides coated with Fluon to prevent escape, and maintained in the laboratory at room temperature. In each tray, tubes of water and 10% sucrose were placed as well as moth pupae and dry dog food.

### Measurement system and stereo image frame

A photograph of the stereo high-speed imaging system for the fire ant alate flight tests is shown in Figure la. The system included a Photron Ultima (www.photron.com) APX camera with a 60-mm Nikon (www.nikon.com) Micro Nikkor lens, a pair of mirrors, a pair of ground glass plates as back lighting screens, and a stereo adaptor between the lens and the high-speed camera. The tested fire ant alates were glued (initially using super glue and later contact cement) on the tip of a fine metal wire (0.3 mm) so that the ant body could be put in a fixed position and the relative motion of the wings could be investigated. As schematically illustrated in Figure lb, in the test section, the tethered fire ant alate was placed between the two mirrors and in front of the backlighting screens, which were illuminated with an Intralux (www.intralux.com) 5100 High Intensity Cold Halogen Light Source through a pair of fiber optic light guides. The stereo adaptor included a cuneiform block and two mirrors. Because of the cuneiform block the fire ant alate could not be directly imaged, whereas the two images of the mirrors in the test section were imaged through the apertures between the inside mirrors and the cuneiform block to the two halves of the image sensor, respectively. In the test the tethered fire ant alate was oriented so as to acquire images of both the side and front views. Once the distances and angles among the image sensor, the lens, the mirrors and the tested fire ant alate were properly setup, the angle between the view directions of the two images was around 90°, and a desired imaging magnification was achieved.

**Figure 2.**
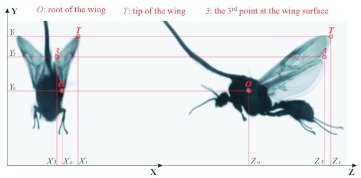
Example of the stereo high-speed image frames and the image coordinates. High quality figures are available online.

The Photron Ultima APX camera was operated at 8000 fps with a resolution of 1024×256 pixels, so that two 512×256 imaging regions could be used for each of the two views. This was sufficient to clearly capture images of flying fire ant alate wings for quantitative analyses. The shadow imaging technique based on back lighting is generally used for high-speed imaging, and it worked extremely well for fire ant studies because the alate wing is transparent, except for the wing veins, so that the shape and structure of the wings can easily be recorded in shadow images. Tests showed that most fire ant alates may fly steadily for a few minutes in the laboratory room with temperature around 27° C when stimulated by a mini blower, and in some cases, the alate flew continuously for hours. The image acquisition took a quarter second for 2,000 frames at 8,000 fps. Microphones were used to record the sound wave to monitor the variation of the wingbeat frequency. The working distance of the stereo imaging system was adjusted according to the size of the tested fire ant alate so that the image-to-object ratio was 22.5 pixel/mm for the males and 20 pixel/mm for the females. Figure 2 shows one of the stereo image frames that was improved with digital image processing tools. As shown in Figure 2, the XY plane information of the fire ant wings is projected to the front view shadow image on the left, and the YZ plane information is projected to side view shadow image on the right. If a wing surface can be assumed as a planar surface, it can be determined with three points indicated in the figure, i.e. the root of the wing (*O*), the tip of the wing (*T*), and the third point that can be chosen at the edge of the wing (*3*). The origin of the image coordinate (*X,Y,Z*) is defined here at the lower left corner of the image frame. The line that connects the root and tip of the wing is defined as wing axis, i.e. *OT*.

**Figure 3.**
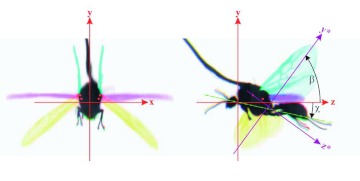
Fire ant wings in a physical coordinate system. High quality figures are available online.

**Figure 4.**
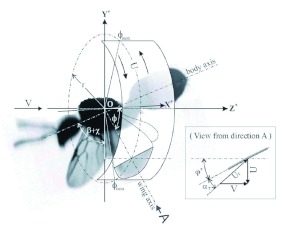
Wing angles in the stroke plane and revolution surface. High quality figures are available online.

As shown in Figure la, two free-field ¼-inch microphones were used to simultaneously record the wingbeat induced sound waveforms from bottom and rear, respectively.

### Wing surface parameters and reconstruction method

Figure 3 and 4 show a few important parameters that are traditionally used for aerodynamic analysis of insect flight (Ellington 1984, Dudley 2002). The longitudinal axis of the fire ant body is in the *yz*-plane, and it forms an angle χ with respect to horizontal axis *oz*. In Figure 3 the blue image indicates the highest position of the forewing tip; the yellow image shows the lowest position of the forewing tip; and the horizontal position of the wings is given by the pink image. The line that is determined from a linear regression of wing tip positions in the side view defines the angle of the stroke plane (*β*). Note that in Figure 3 the origin of the physical coordinate (*x,y,z*) is defined at the center of the neck of the ant body. The root position of a fire ant wing marked with red dot in Figure 3 can be estimated with the joint point of wing axis lines of deferent image frames. In order to determine parameters that may be used for an aerodynamic analysis, the coordinate system (x,y,z) is rotated and shifted to (x* ,y* ,z*), so that x*y* -plane overlaps with the stroke plane and the root of the forewing is on z*-axis. As shown in Figure 4, the angular position in the stroke plane 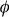 and the wing angle in the revolution surface φ* are determined, and the wingbeat angular amplitude is obtained as *Φ*=(*
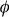
_max_* - *
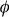
_min_*). Other parameters shown in Figure 4 include air flow velocity ***V***, revolving velocity ***U***, relative velocity ***U_r_*** and attack angle α_τ_. An aerodynamic analysis cannot be completed until the magnitude of V is determined with flow measurement or CFD simulation.

**Figure 5.**
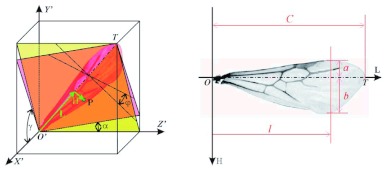
Wing surface Parameters: (left) wing position and orientation in local coordinate system (*X'*,*Y'*,*Z'*), and (right) structure details in wing surface coordinate system (*L*, *H*). High quality figures are available online.

Figure 5 left shows the coordinates and parameters for determining a single wing surface marked with pink color. The wing surface passes through the coordinate origin that is set at the root of the wing, and it has angle γ when cut with XY-planes and α when cut with XZ-planes, i.e. γ is the wing surface angle in the front view and α in the side view. With these two angles, the wing surface can be represented in local coordinate system (*X',Y',Z'*) as: γ *X*′ - *Y*′ + tan α · *Z*′ = 0. Another way to represent the wing surface is to use wing axis *OT* and the wing rotational angle φ that is the angle between the wing surface and the reference surface (marked with yellow in Figure 5), which is determined with wing axis *OT* and the coordinate axis *OZ*. For a wingbeat with a constant frequency, the wingtip position and the above mentioned angles are functions of the phase that is represented here as *t/T*, wherein *T* is the wingbeat period and *t* is the time in the wingbeat period. The start of the wingbeat period (i.e. *t=0*) is defined when the wingtip is around the maximal y-position and rotational angle φ equals zero. As shown in Figure 5 right, a high resolution digital image of the tested fire ant wing is used to provide detailed information of the wing structure with wing surface coordinate system (*L*, *H*). The shape of a fire ant wing is determined with trailing edge width *a*, leading edge width *b* at position *l* on the wing axis, the length of the wing is recorded as *C*.

Assume that the wing surface parameters in Figure 5 left are determined and the high-resolution wing image in Figure 5 right has gray value distribution *G(L,H)*. For an arbitrary point on the wing surface *P*, which is determined with coordinates (*x'*,*y'*,*z'*), the distance from *P* to the wing axis *OT* can be determined as *H*, and the projected length of *OP* on axis *OT* can be determined as *L*. Then the gray value of the three dimensional image at point *P* is obtained as *g(x'* ,*y'* ,*z'*) = *G(L,H)*. Since the coordinates *x'* ,*y'* ,*z')* have only two independent variables, the wing images projected to XY-plane (front view), YZ-plane (side view) and XZ-plane (top view) can be determined as *g(x' ,y'), g(y' ,z') and g(x' ,y')*. When the stereo images of both the forewing and hindwing are reconstructed, they can be assembled according to the wing root positions as shown in Video 3 and 4.

## Results

While tests were conducted with many male and female fire ant alates, only data sets for those alates that appeared normal in their flight behavior have been kept in the data base. The high-speed video of each test case was inspected to exclude data sets that indicated defective wings, defective or missing body parts, interfered wing motions, unbalanced wing motions, non-periodical wing motions, and other behaviors that were considered abnormal. Image recordings of 9 female and 9 male black imported fire ant alates were considered as valid. Each of these image recordings consisted of more than 2000 frames, and the time interval between frames was 1/8000 second. In the tests the slowest wing motion had a frequency of 81 Hz, so that at least 20 wingbeat periods were recorded in the one quarter second imaging acquisition time. Test data demonstrated a constant wingbeat period for each test case.

**Figure 6.**
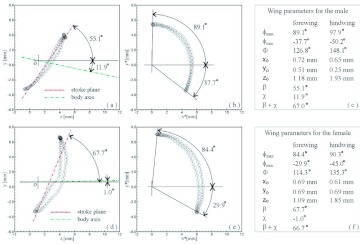
Wingbeat parameters of the selected fire ant alates: (a) forewing tip positions in yz-plane for the male fire ant, (b) forewing tip positions in the stroke plane for the male fire ant, (c) summary of wing parameters for the male, (d) forewing tip positions in yz-plane for the female fire ant, (e) forewing tip positions in the stroke plane for the female fire ant, (f) summary of wing parameters for the female. High quality figures are available online.

**Figure 7.**
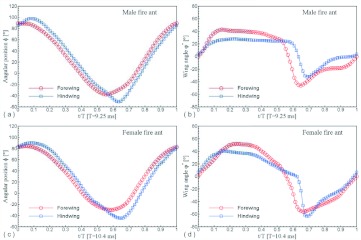
Wing motion data in the stroke plane and revolution surface: (a) angular positions of the male fire ant wings; (b) wing angles in the revolution surface of the male fire ant wings; (c) angular positions of the female fire ant wings; (d) wing angles in the revolution surface of the female fire ant wings. High quality figures are available online.

A representative male and a representative female fire ant alate (*S. richteri*) were selected for detailed data analyses. The male live weight was 5.5 mg and the female live weight was 11.4 mg. Their respective body lengths were 6.2 mm and 8.3 mm, while their wingbeat frequencies were 108 Hz and 96 Hz. Some wingbeat parameters were obtained for these two alates by evaluating the high-speed image frames (Figure 6). Figure 6c and 6f show that the wingbeat angular amplitude (*Φ*) of the male was about 10% larger than that of the female for both the forewing and the hindwing, and that the angular amplitude of the hindwing was around 15% larger than that of the forewing for both the male and the female. Since the fire ant alates were tethered during the tests, the measured stroke plane angle *β* and the body angle χ may be different from those during natural flight, however, the sum of these two angles (i.e. *β*+*χ*) may not be affected by the tethering. The tests show that the angle between the wingbeat stroke plane and the longitudinal axis of the fire ant body was almost the same (i.e. 67.0° & 66.7°) for both the male and the female. The wing root position (*x_0_*, *y_0_*, *z_0_*) was determined in the physical coordinate system as shown in Figure 3, which is necessary for reconstructing the stereo image frames. The wingtip coordinate and wing surface angle distributions in the wingbeat period are given in Table 1 and 2 for the male and female fire ant alate, respectively. The angular position distributions in the stroke plane and the wing angles in the revolving surface are shown in Figure 7 for the selected fire ant alates.

**Figure 8.**
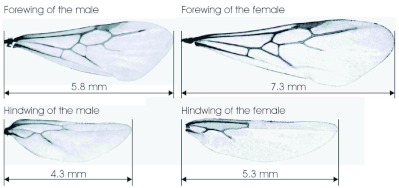
Size, shape and structure of wings for the selected *S. richteri* alates. High quality figures are available online.

Since the stereo high-speed image frames were obtained with relatively low digital resolutions, i.e. 22.5 pixel/mm for the male and 20 pixels/mm for the female, shadow images of both the forewings and hindwings were taken with a ratio of 128 pixel/mm after the stereo imaging tests, and they are shown in Figure 8. The high resolution wing images in Figure 8 provide not only the dimension and shape of the wings but also the structural details of the wing veins. Detailed data of the wings are given in Table 3.

With the wingbeat parameters, wingtip position distributions, wing surface angle distributions, and wing structure images given above, stereo image frames were numerically reconstructed. In Video 1 (male alate) and Video 2 (female alate) the reconstructed stereo wing images (below) were compared with those recorded in the stereo high-speed imaging tests (above) in 74 (male) and 83 frames (female). Since the view angles of the two cameras were perpendicular, there was no problem determining the three dimensional position of a visible marker (e.g. wingtip and wing root) on the wing surface. It would be an ideal test case when the body axis of the tested fire ant alate is in the YOZ-plane. In most cases, however, there was a small deviation angle between the fire ant body axis and YOZ-plane, so that the side view images of the two forewings and two hindwings overlapped incompletely. The angular deviation could be calculated according to the difference between the left and right forewing data. In the cases of Video 1 and Video 2, the angular deviations were determined to be 4° and 5°, respectively. The angular deviations were considered in the reconstructed wing images. The animated frames in the videos demonstrate that the reconstructed stereo wing images match the captured stereo high-speed video images very well, however, slight deviations could be observed at t/T=0.08∼0.16 when the two hind wings touched together, and t/T=0.56∼0.68 when the forewings rotated very fast. The three view reconstructions of the selected male and female fire alate are illustrated with Video 3 and Video 4, respectively.

**Table 1.**
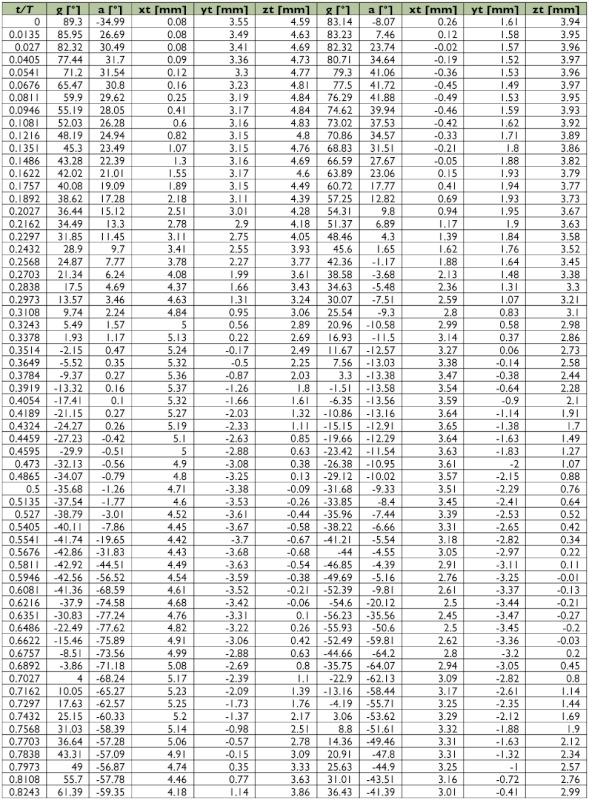
Wing motion details of the selected male fire ant alate.

**Continued.**
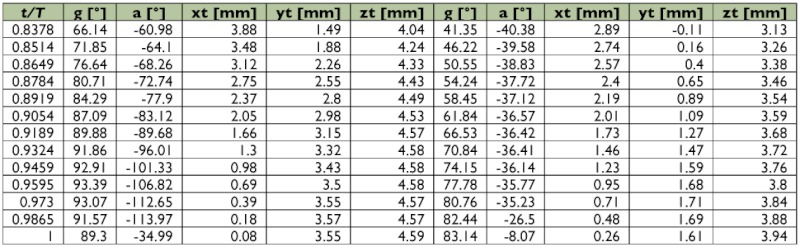

**Table 2.**
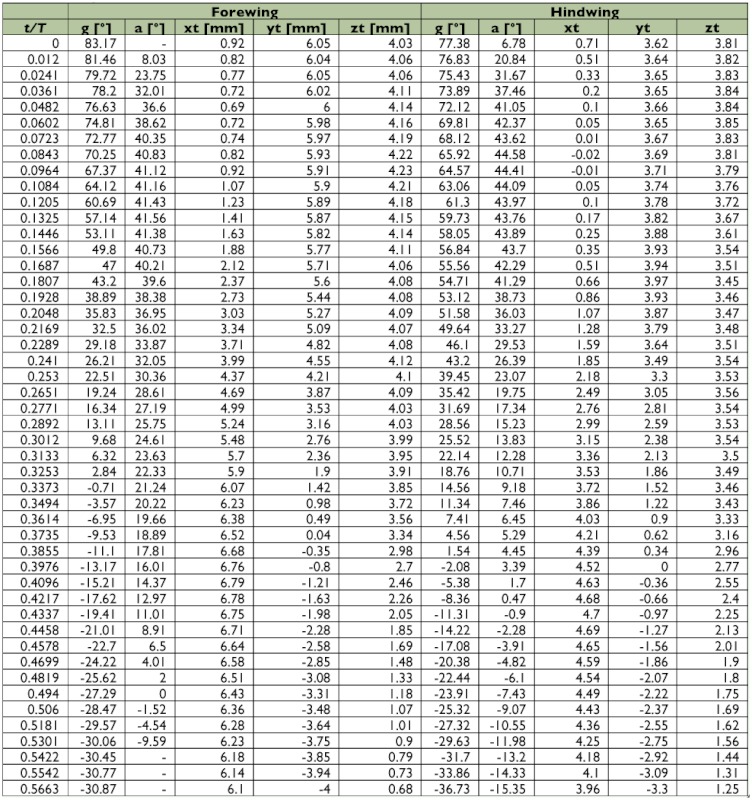
Wing motion details of the selected female fire ant alate.

**Continued.**
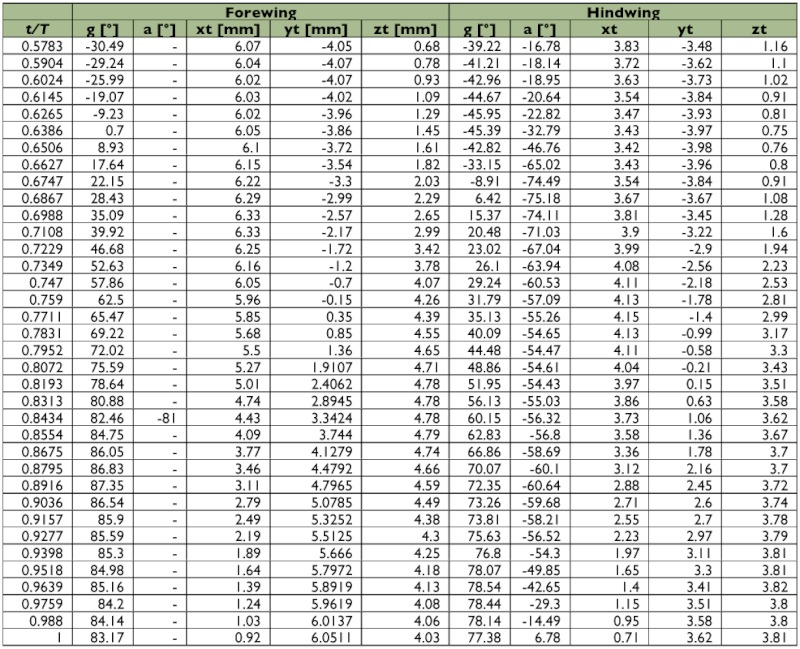

**Video 1.**
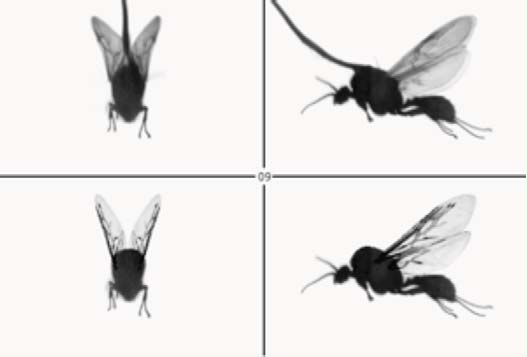
Comparison between the reconstructed wing images (below) and the original stereo wing images (above) for the male (*S. richteri*) alate (74 frames in one period). Videos are available online.

**Video 2.**
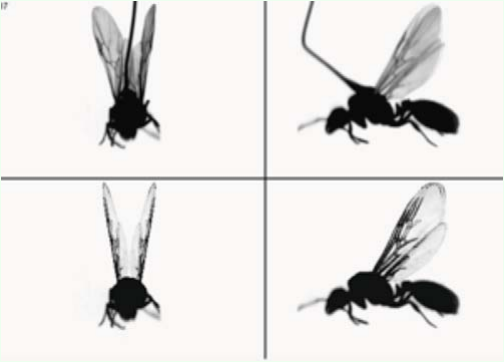
Comparison between the reconstructed wing images (below) and the original stereo wing images (above) for the female fire ant alate (83 frames in one period). Videos are available online.

**Video 3.**
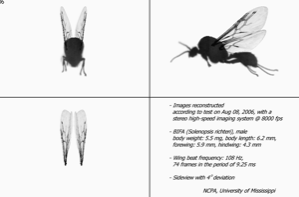
Three-dimensional view reconstruction of the male fire ant alate wingbeat in one period. Videos are available online.

**Video 4.**
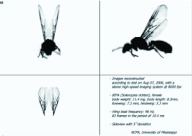
Three-dimensional view reconstruction of the female fire ant alate wingbeat in one period. Videos are available online.

**Figure 9.**
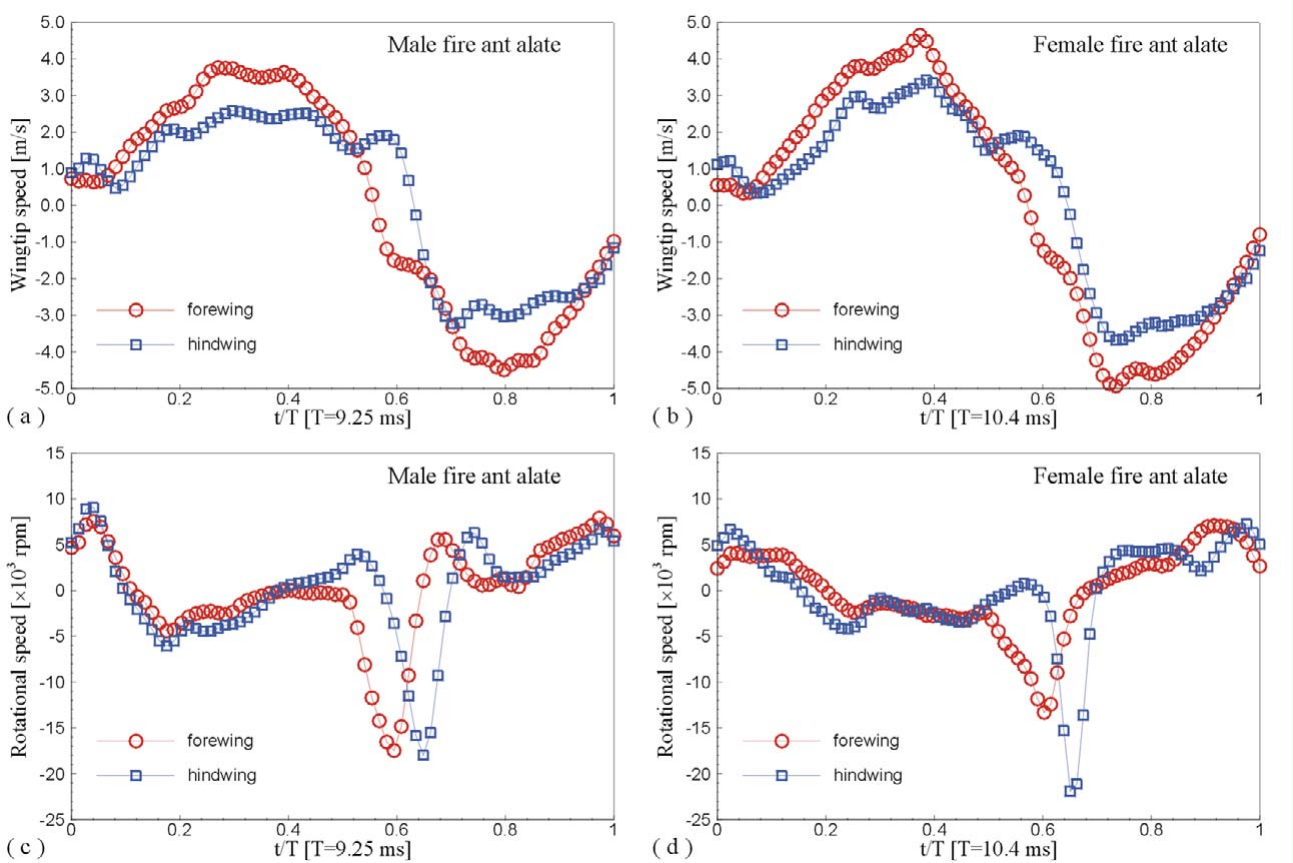
Wingtip speed and wing surface rotational speed of the selected *S. richteri* alates: (a) wingtip speed of the male; (b) wing rotational speed of the male; (c) wingtip speed of the female; (d) wing rotational speed of the female. High quality figures are available online.

The data presented in Figure 6, Table 1, 2 and 3 provide inputs for a mathematical description that can easily be used to build a numerical model for CFD simulation. Further deductions of the data can be used to explain some aerodynamic or aeroacoustic phenomena. For example, the periodical distributions of the wingtip speed and wing surface rotational speed were deduced from the data and are shown in Figure 9, which shows that, for the forewing and the hindwing of both the male and the female, the maximal wingtip speed of the downwards wing motion was around t/T=0.35, whereas the maximal upward wingtip speed was around t/T=0.75.

**Table 3.**
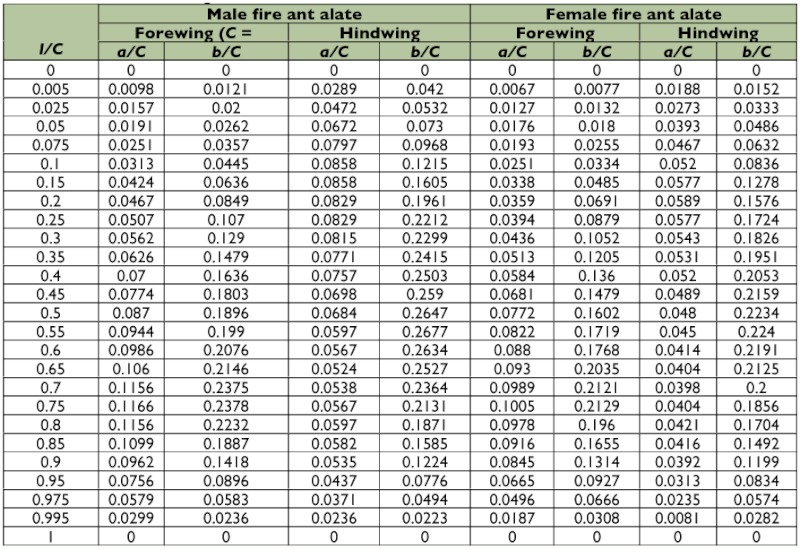
Geometrical data of wings for the selected fire ant alates.

**Figure 10.**
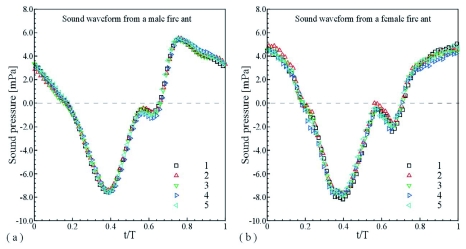
Overlapped sound waveforms measured from the bottom of the *S. richteri* alates: (a) sound pressure of 5 periods from a male, (b) sound pressure of 5 periods from a female. High quality figures are available online.

The wing surface rotational speed had negative high amplitude peaks between t/T=0.5 and 0.6. The sound waveforms measured about 10-mm away from the bottom of a male and a female fire ant alate are shown in Figure 10, in which the sound pressure distributions of five periods overlapped. The sound pressure had a minimum at t/T≈0.36 for both the male and female case, which should result from the maximal downward wingtip speed. In the male fire ant case, the sound pressure went up to maximum at t/T≈0.75 that may be related to the maximal upward wingtip speed. It seems that the high-speed rotation of the fire ant wings between t/T=0.5 and 0.7 generated a negative pressure pulse to inhibit the sound pressure increase and formed a local minimum at t/T≈0.65. In the female case, since the negative rotational speed of the hindwing was much higher than the forewing of the female and both the forewing and hindwing of the male, the sound pressure increase was greatly inhibited, so that no maximum could be seen around the maximal upward wingtip speed.

## Discussion

The stereo high-speed imaging system applied a simple optical configuration to avoid a much more expensive two-camera system for taking stereo videos of fire ant alate wingbeat motion. Unlike the usual stereo imaging adaptors, in our optical configuration the focus planes of the two views are perpendicular to each other, so that the three dimensional positions of the fire ant alate wings can be directly read from the stereo image pairs. With post-processing of the image the background noise is removed and clear images are obtained. The basic requirement of the camera lens is that the undistorted imaging area width should not be less than double that of the image sensor width. In addition, there should be enough room between the camera lens and the image sensor to install a pair of mirrors. With the 60 mm lens, the field of view of the presented stereo image system is around 24×12 mm^2^ that is sufficient to image the largest fire ant alate. When the working distance and the outside mirror angles are adjusted, this system can be used for larger or smaller winged insects.

In this work the flight parameters and wing motion details of a male and a female fire ant alate were completely described, so that the three-dimensional wing motions could be numerically reconstructed. Based on the twoview image frame from the stereo high-speed imaging test, wing images of arbitrary view direction were obtained with numerical reconstruction, e.g. the top-view images in Video 3 and Video 4. When the thickness and proper profile of fire ant wings are considered, the obtained fire ant alate flight data can be used for a further computational fluid dynamic (CFD) analysis.

Further deductions of the fire ant alate flight data can be used to explain some aerodynamic or aeroacoustic phenomena. For example, the wingtip speed and wing surface rotational speed were used to explain some waveform features of the wingbeat-induced near field sound.

In this work a fire ant wing was assumed to have a planar surface, so that it could easily be determined with the coordinates of three points that could be read from the stereo image frame. This assumption works very well in 80% of the wingbeat period. The animated frames in Video 1 and Video 2 show that the two hindwings were obviously bent when they touched together within *t/T*=0.08∼0.16, and that the forewings were slightly deformed when they rotated rapidly during *t/T*=0.56∼0.68. A higher order surface fit may be necessary to include the wing surface bending and deformation in the data, however, this would require a much more complicated algorithm and a higher digital resolution of the imaging system. It should also be mentioned that the flight of tethered fire ant alates may differ from the flight of free flying alates.

## Abbreviations

***a***: trailing edge width;
***b***: leading edge width;
***C***: wing length;
**CFD**: computational fluid dynamics;
**fps**: frames per second;
**FW**: forewing;
**HW**: hindwing;
***I***: position at wing axis;
***L, H***: wing surface coordinates;
***OT***: wing axis;
**rmp**: revolutions per minute;
***t***: time in wingbeat period;
***T***: wingbeat period;
***U***: revolving velocity;
***U_r_***: relative velocity;
***V***: air flow velocity;
***X, Y, Z***: stereo image coordinates;
***x, y, z***: physical coordinates;
***X',Y',Z'***: local coordinate;
**α**: side view wing surface angle;
**α_τ_**: attack angle;
***β***: wingbeat stroke plane angle;
***γ***: front view wing surface angle;
******: angular wing position;
***φ***: wing rotational angle;
***Φ***: wingbeat angular amplitude;
**φ***: wing angle in revolution surface;
***_max_***: maximal angular position;
***_min_***: minimal angular position;
***χ***: body orientation angle
